# Supplementary material for: Face-to-Face Assembly of Ag Nanoplates on Filter Papers for Pesticide Detection by Surface-Enhanced Raman Spectroscopy
Source: Nanomaterials (Basel). 2022 Apr 19;12(9):1398. doi: 10.3390/nano12091398 (PMC9104380; doi:10.3390/nano12091398)
Supplement: Supplementary file 1 [file nanomaterials-12-01398-s001.zip › nanomaterials-1652966-supplementary.pdf]

Supporting Information

Face-to-face Assembly of Ag Nanoplates on Filter Papers for Pesticide Detection by Surface-enhanced Raman Spectroscopy

Sulin Jiao[‡1,2], Yixin Liu[‡2], Shenli Wang[‡3] Shuo Wang[1], Fengying Ma[2], Huiyu Yuan[4], Guangchao[*2], Zheng Yuan Zhang[*2], Kun Dai[*1], and Chuntai Liu[1]

[1]School of Materials Science and Engineering, Key Laboratory of Materials Processing and Mold (Zhengzhou University), Ministry of Education; Henan Key Laboratory of Advanced Nylon Materials and Application (Zhengzhou University), Zhengzhou University, Zhengzhou, 450001, P.R. China

[2]School of Physics and Microelectronics, Key laboratory of Material Physics, Ministry of Education, Zhengzhou University, Zhengzhou, 450001, P.R. China.

[3]School of Food Science and Engineering, Henan University of Technology, Lianhua Road 100, Zhengzhou, 450001, P.R. China

[4]Henan Key Laboratory of High Temperature Functional Ceramics, School of Materials Science and Engineering, Zhengzhou University, Zhengzhou, 450001, P.R. China.

Email: zhengguangchao2008@gmail.com (G. Zheng), yzhuaudipc@zzu.edu.cn (Y. Zhang), kundai@zzu.edu.cn (K. Dai)

* Corresponding author

‡ Equal contributors

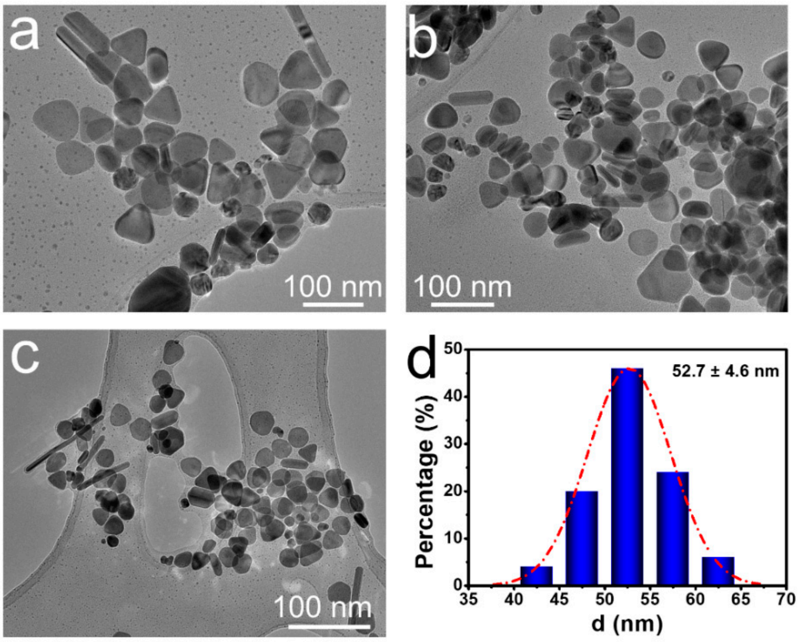

Figure S1. (a-c) TEM images of silver nanoplates, (d) size distribution for silver nanoplates.

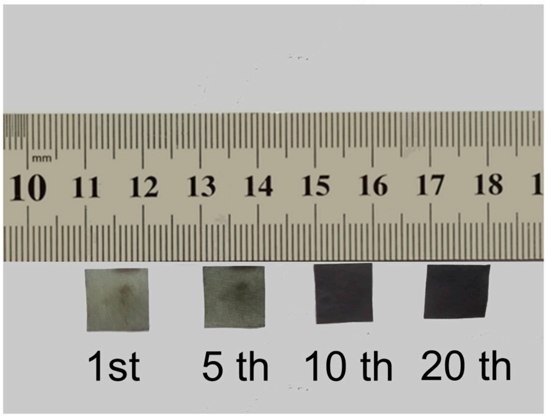

Figure S2.Photos of Ag nanoplates coated filter paper (1 st-20 th).

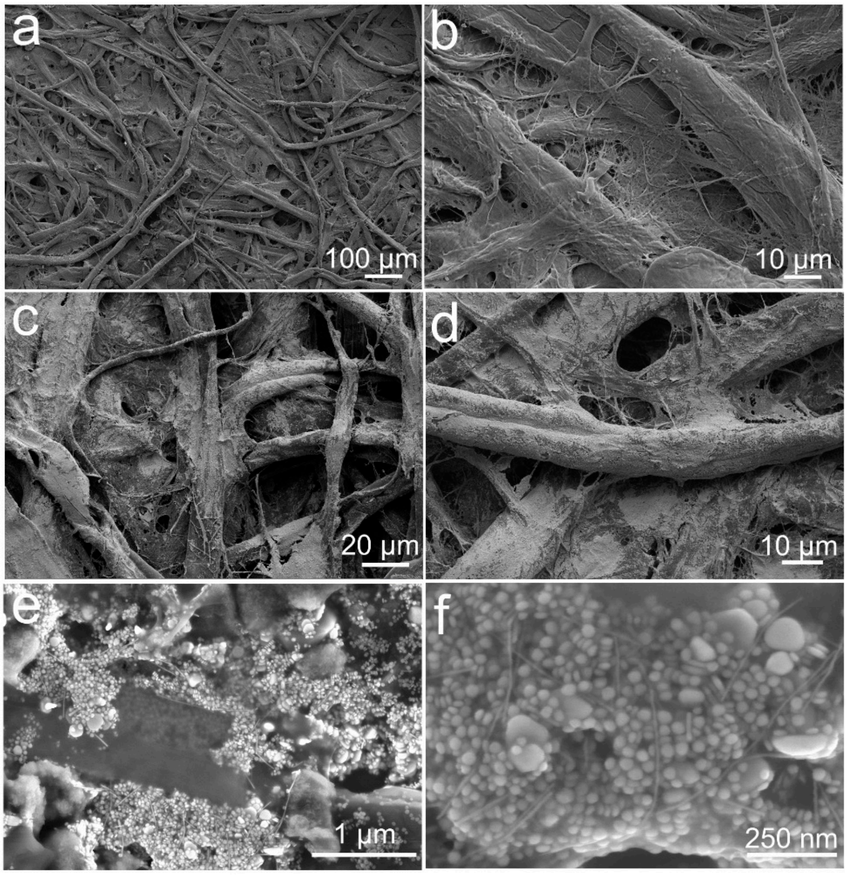

Figure S3. (a-b) SEM images of pure filter paper. (c-f) SEM images of Ag nanoplates coated filter paper.

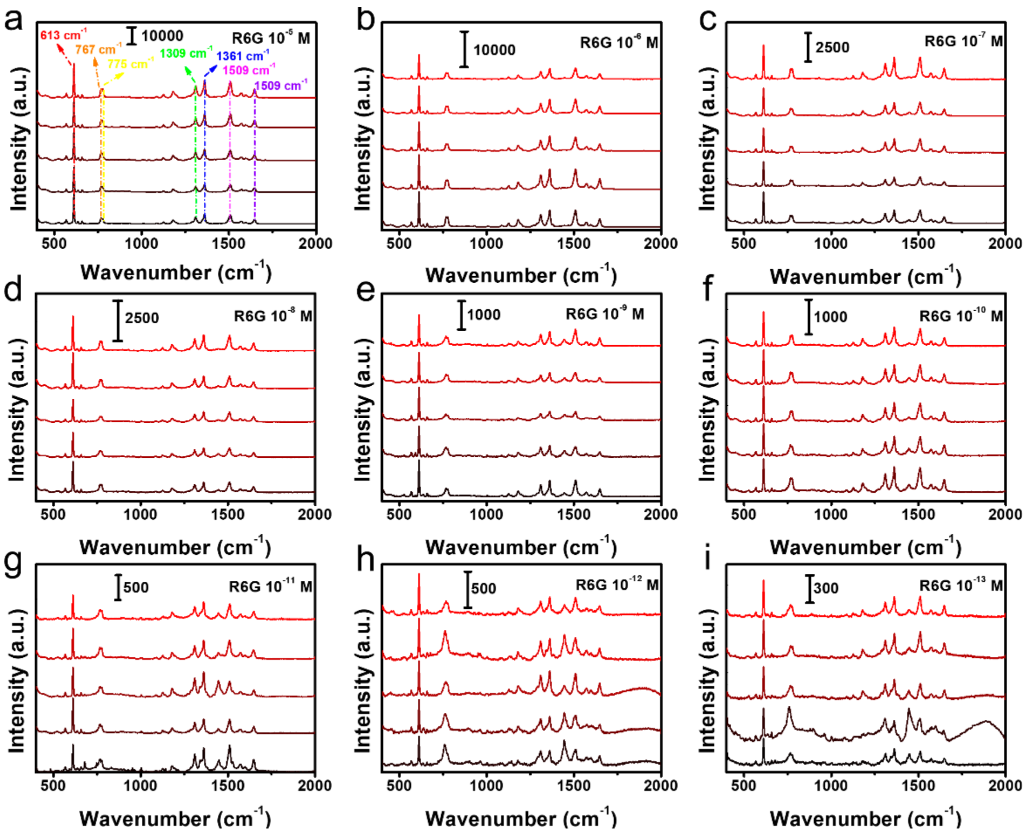

Figure S4. (a-i) Raman spectra of different concentrations of R6G (10^{-5} M -10^{-13} M). In (a), the main Raman active vibrational modes are marked.

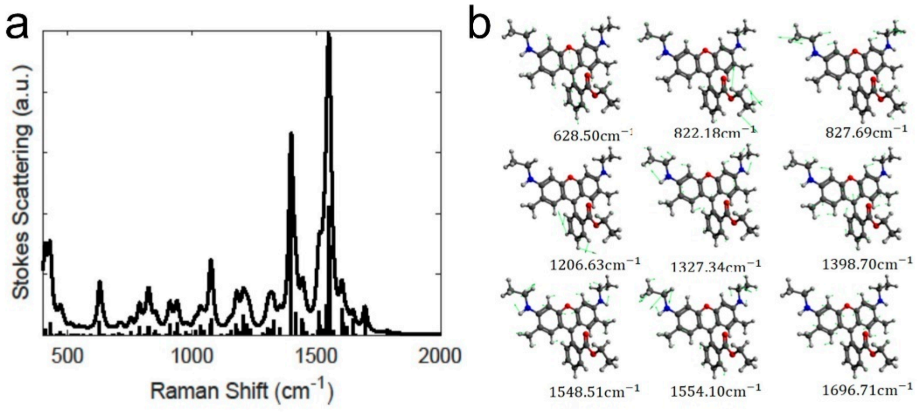

Figure S5. (a) Simulated Raman spectra of R6G molecules (for laser excitation 633 nm, solid curve) obtained by broadening the sticks for individual vibrational modes with Lorentizian function of a width of 20 cm^{-1}. (b) The vibrational pattern of the selective Raman active vibrational modes, where white, gray and blue spheres are the hydrogen, carbon and nitrogen atoms, respectively.

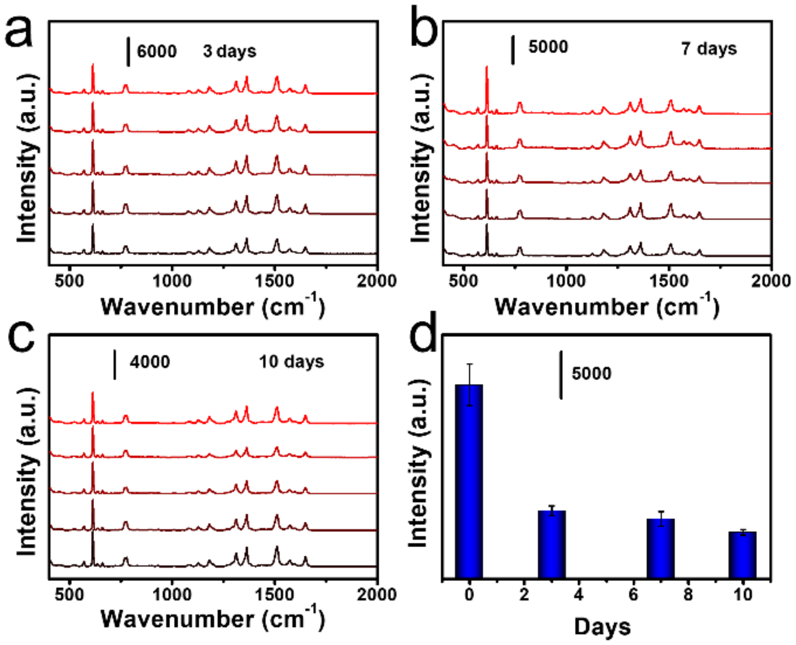

Figure S6. The Raman signal of filter papers stored at room temperature for 3 days (a), 7 days (b), 10 days (c). (d) Raman intensity histogram of substrates.

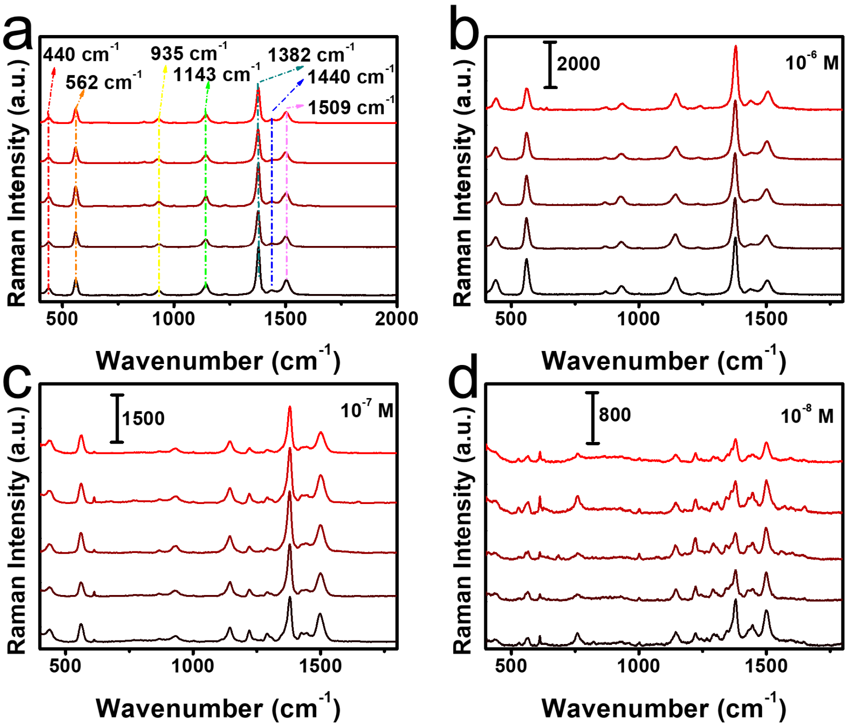

Figure S7. (a-d) Raman spectra of different concentrations of thiram in acetone (10^{-5} M -10^{-8} M). In (a), the main Raman active vibrational modes are marked.

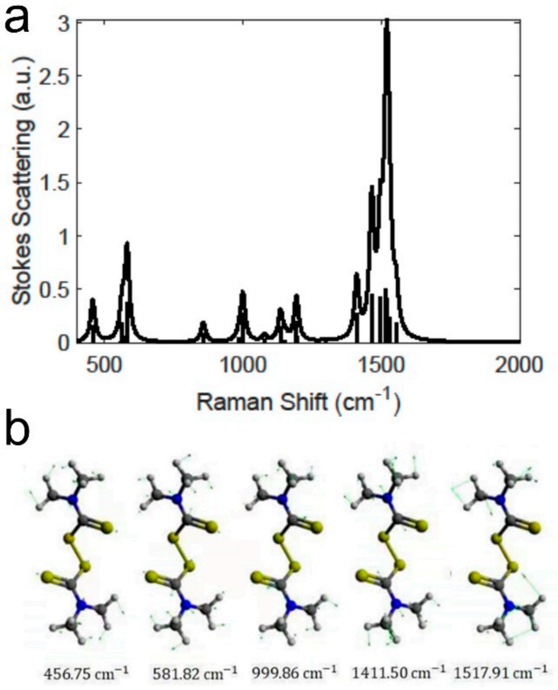

Figure S8. (a) Simulated Raman spectra of thiram molecules. (e) The vibrational pattern of the vibrational modes, where the white, gray and blue spheres are the

hydrogen, carbon and nitrogen atoms, as before, and the yellow and red spheres are sulfur and oxygen atoms.

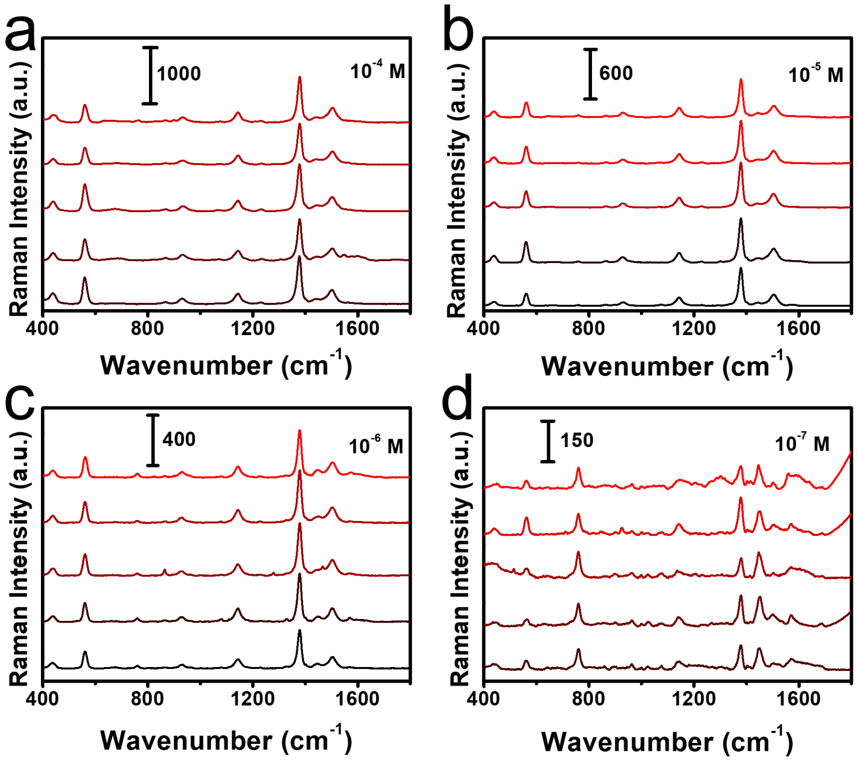

Figure S9. (a-d) Raman spectra of different concentrations of thiram in juice (10^{-4} M - 10^{-7} M).

Table S1. Comparison of other substrates.

Articles	Materials	Methods	LOD
Hasi et al. [36]	Ag NPs coated filter paper	Deposition	4-MBA 10^{-8} M
Wang et al. [29]	Ag NPs coated filter paper	Immersed into the colloid	R6G 5×10^{-8} M Thiram 4.6 ng/cm^2
Zhang et al. [50]	Ag NPs coated filter paper	Vacuum filtration	BPA 0.005 ng/mL
Moram et al. [51]	Ag/Au NPs coated filter paper	Immersed into the colloid	MB-5 nM、PA-5 μM、DNT-1 μM and NTO-10 μM
Sun et al. [31]	Core-shell Ag@SiO_2 NPs coated filter paper	Dripped onto paper substrate	Thiram 1×10^{-9} M
Zhu et al. [52]	Au@Ag NPs coated filter paper	printing	LOD of Thiram is 1×10^{-9} M
He et al. [53]	Au NSs coated filter paper	Dripped onto paper substrate	LOD of CV is 1 nM
Pagano et al. [54]	Ag nanodisks decorated filter paper	/	LOD of TC is 10^{-9} M
Tegegne et al. [55]	1T-MoS_2/AgNCs nanocomposite	Ultrasonicated	LOD of Thiram is 0.62 nM
Zhu et al. [56]	AgNC−rGO Sponge	Pongelike hybrid was immersed into colloid	LOD of Thiram is 44 nM
Zhu et al. [35]	Ag-nanocubes/graphene-oxide/Au-nanoparticles	Sputtered	LOD of Thiram is 1 nM
Xiong et al. [30]	Au NPs coated cellulose fiber	Deposited of CNF/AuNP	LOD of Thiram is 1 nM
Zhang et al. [34]	Micropyramid Array and Silver Nanoparticles	Growth on substrates	LOD of Thiram is 10^{-7} M

Table S2 The comparion of LOD of Thiram with other works.

Authors	Substrates	LOD of Thiram
Xu et al. [57]	Ag decorated α-Fe_2O_3 coated SiO_2 fabric	1×10^{-6} M
Zhang et al. [34]	Ordered Micropyramid Array and Silver Nanoparticles	1×10^{-7} M
Zhu et al. [56]	Ag NCs/rGO sponge	4.4×10^{-8} M
He et al. [53]	Au NSs coated filter paper	1×10^{-9} M
Our work	Ag nanoplates coated filter paper	1×10^{-8} M

Table S3 Assignment of SERS peaks to the vibrational modes

Rhodamine		Thiram	
SERS Peaks	Vibration mode	SERS Peaks	Vibration mode
613 cm^{-1}	in-plane deformation of the xanthene ring	437 cm^{-1}	C-S stretching vibration
767 cm^{-1}	C-H out-of-plane bend of	589 cm^{-1}	S-S stretching vibration
775 cm^{-1}	the xanthene ring	933 cm^{-1}	CH_3-N stretching vibration
1309 cm^{-1}		1142 cm^{-1}	C-N stretching vibration
1361 cm^{-1}	aromatic C-C stretching	1377 cm^{-1}	deformation of CH_3
1509 cm^{-1}	vibration	1502 cm^{-1}	C-N stretching vibration
1647 cm^{-1}			
